# Factors associated with family caregiver burden among frail older persons with multimorbidity

**DOI:** 10.1186/s12877-022-02858-2

**Published:** 2022-02-28

**Authors:** Teck Yong Gabriel Ding, Jacqueline Giovanna De Roza, Cheuk Ying Chan, Poay Sian Sabrina Lee, Sin Kee Ong, Kaiwei Jeremy Lew, Hui Li Koh, Eng Sing Lee

**Affiliations:** 1 Geylang Polyclinic, National Healthcare Group Polyclinics, Singapore, Singapore; 2 Woodlands Polyclinic, National Healthcare Group Polyclinics, Singapore, Singapore; 3 Clinical Research Unit, National Healthcare Group Polyclinics, Singapore, Singapore; 4 Lee Kong Chian School of Medicine, Nanyang Technological University, Singapore, Singapore

**Keywords:** Caregiver burden, Frail, Multimorbidity

## Abstract

**Introduction:**

Singapore is facing an ageing population and the care needs of the population will increase in tandem. A segment of this population would be living with multimorbidity and frailty. Frailty is defined as an age-related state characterised by reduced strength and physiologic malfunctioning. Multimorbidity refers to the coexistence of multiple chronic conditions in an individual. Older adults are more likely to have frailty and multimorbidity, and this would increase the burden of their caregiver. Our study aimed to determine the prevalence of caregiver burden for primary family caregivers of frail older adults with multimorbidity. We also investigated the factors that were associated with primary family caregiver burden.

**Methods:**

This was an interviewer-administered, cross-sectional study of primary family caregivers of frail older patients with multimorbidity that was conducted in two National Healthcare Group polyclinics. Convenience sampling was used. The 12-item Zarit Burden Index (ZBI) was used to assess primary family caregiver burden. The scores of the ZBI range from 0 to 48, with a score of 10 or above indicating that the primary family caregiver perceives burden. Descriptive statistics were used to provide information regarding the caregivers and the care recipients. Multivariable logistic regression was used to investigate the factors affecting primary family caregiver burden.

**Results:**

One hundred eighty-eight family caregivers were interviewed and 71.8% of them perceived burden on the ZBI. 59.6% were caregivers to their parents and 18.1% of them had multimorbidity. Almost two-thirds of the caregivers interviewed were female. After adjusting for other factors via multivariable analysis, the ethnicity of the caregiver and the increase in time spent caregiving per week were the two factors positively associated with family caregiver burden. A Chinese primary family caregiver had almost three times the odds of perceiving burden when compared to a non-Chinese primary family caregiver.

**Conclusion:**

Caregiver burden was high amongst primary family caregivers of frail older adults with multimorbidity. Being a Chinese primary family caregiver compared to non-Chinese ethnic groups as well as being a primary family caregiver who spent increased time caregiving per week were the two factors positively associated with family caregiver burden. Further exploratory, qualitative studies can be done to find out the reasons to Chinese primary family caregivers being more burdened compared to the non-Chinese primary family caregivers. In addition, the specific factors related to increased time caregiving per week and family caregiver burden can also be studied.

## Introduction

By 2030, one in four Singaporeans will be over 65 years of age and the care needs of the population will increase in tandem [1]. As a result, the old-age support ratio will decrease from 4.8 today to 2.7 in 2030, and caregiving burden will grow [1]. Based on 2015 census of the central region in Singapore, more than two-thirds of older adults aged 60 years and above are either living with illness (57.6%) or frailty (14.1%) [2]. A study in Singapore also found that a high percentage of older adults aged 60 years and above who are living with illness tend to have multimorbidity [3]. Older adults are more likely to have frailty and multimorbidity, and this would increase the burden of their caregiver.

Frailty is defined as an age-related state characterised by reduced strength and physiologic malfunctioning [4]. Frail persons are at increased risk of adverse outcomes from relatively minor stressors and more susceptible to dependency, vulnerability, and death [4]. Multimorbidity refers to the coexistence of multiple chronic conditions in an individual [5]. A study in Canada found that about half of patients attending primary care practices had multimorbidity [5]. Multimorbidity was associated with substantially higher healthcare utilisation and social care costs among older adults in Singapore [6]. Frailty and multimorbidity are intricately linked. A large scale study in the United Kingdom found significant association between frailty and multimorbidity [7]. The National Institute for Health and Care Excellence (NICE) and British Geriatrics Society emphasise the importance of recognising frailty to identify patients with multimorbidity who are at greater risk of adverse outcomes, including caregiving burden. Frailty and multimorbidity both have been proven to be a predictor and an outcome of each other, as well as to be both predictors of disability and mortality [8].

Despite the availability of formal care services, the primary caregiving role for older adults still falls on informal family caregivers in Singapore [9]. In Singapore, a primary informal caregiver is defined as a family member or friend (but not a foreign domestic worker) most involved in providing care or ensuring provision of care [10]. To our knowledge, there were no studies found on caregiver burden for primary family caregivers of older adults with both frailty and multimorbidity.

We used The Informal Caregiving Integrative Model (ICIM), proposed by Gérian et al. as a theoretical framework on exploring the determinants of caregiver burden [11].

Our study aimed to determine the prevalence of caregiver burden for primary family caregivers of frail older adults with multimorbidity. We explored the association between the frailty status of care recipients and the burden experienced by their primary family caregivers. We also investigated the association between the number of chronic conditions in the care recipient with multimorbidity and the burden experienced by his/her primary family caregiver. Finally, we studied the association of the other factors that can affect caregiver burden in these caregivers.

The findings and knowledge gained from our study would be pertinent for developing interventions to alleviate burden experienced by caregivers of this group of frail older adults with multimorbidity.

## Methods

### Study design and setting

This was an interviewer-administered cross-sectional study that was conducted in two National Healthcare Group polyclinics from July to December 2020. A polyclinic is a health care centre that provides subsidised primary care, which includes primary medical treatment, preventive healthcare, and health education. Convenience sampling was used in this study.

All eligible caregiver-care recipient dyads who turned up for medical appointments in the clinics were invited to participate.

Eligible caregivers were interviewed by trained members of the study team using a questionnaire who collated the data via the Research Electronic Data Capture (REDCap®) (intranet accessible platform). REDCap® is a web-based application developed by Vanderbilt University to capture data for research studies in a systematic manner [12].

### Participants

#### Care recipients

To be selected for the study, we first identified suitable care recipients who fulfilled these criteria – 65 years and above, had three or more chronic medical conditions, scored 4 and more on the Clinical Frailty Scale. They were neither institutionalised, actively receiving chemotherapy nor receiving palliative care with projected life expectancy of less than 6 months.

#### Caregivers

We next identified eligible participants who were caregivers of the suitable care recipients if they fulfilled the following criteria - primary family caregivers (spouse, child or other relative) most involved in providing direct care or ensuring provision of care for the care recipient for at least 3 months, were not receiving a salary for caregiving and were able to communicate in English, Mandarin, Malay, or Tamil.

### Ethics approval and consent to participate

The study was granted ethics approval by the National Healthcare Group Domain Specific Review Board (Reference: 2020/00014). Informed consent was obtained from all recruited participants.

### Outcome variables

The questionnaire comprises of three parts, namely the characteristics of caregivers, the characteristics of care recipients and the 12-item Zarit Burden Scale (ZBI) to assess caregiver burden. The scores of the ZBI range from 0 to 48, with a score of 10 or above indicating that the family caregiver perceives burden [13].

The use of the 12-item ZBI was extended and validated in other caregiver populations such as informal caregivers of elderly persons irrespective of level of cognition (Cronbach’s alpha 0.90) [14] and caregivers of community-dwelling older adults with diverse comorbidities (Cronbach’s alpha 0.81) [15]. The 12-item ZBI was also used and validated locally, but for caregivers of persons with dementia [16]. It has been used to assess caregiver burden in frail older persons in larger studies overseas [13, 17]. The 12-item ZBI showed good construct validity and internal consistency where overall Cronbach’s alpha was 0.88. Correlations between the short and the full version ranged from 0.92 to 0.97 [18].

### Independent variables

The 9-point Clinical Frailty Scale (CFS) was developed by Rockwood et al. as a tool to assess frailty in clinical practice [19]. Studies found that the CFS was highly correlated (r = 0.80) with the Frailty Index. The CFS comprised of nine categories that broadly summarise the overall level of fitness and function of an older adult. The CFS is a visual and written chart with nine graded pictures. Care recipients being eligible for this study were in categories 4 (vulnerable) and above.

We defined multimorbidity as the coexistence of three or more chronic conditions in an individual, based on Fortin et al.*’s* list of conditions which has been found to be the most suitable for describing multimorbidity in the Singapore primary care setting [20]. Using three or more chronic conditions for defining multimorbidity as the higher cut-off threshold identified a smaller number of patients with higher needs compared to using two or more chronic conditions*.* The number of chronic conditions in an individual with multimorbidity is presented and analysed as a continuous variable.

### Sample size

The ‘rules of thumb’ for determining sample size for regression equations using six or more predictors states that an absolute minimum of 10 participants per predictor variable is appropriate [21].

Hence, for 18 independent variables, we needed a minimum sample size of 180 participants.

### Validity and reliability

A pilot study of 20 participants was performed to assess the face validity and test-retest reliability of the questionnaire. The questionnaire was understood clearly without the need for amendments.

The intraclass correlation (ICC) was performed for test-retest reliability for the overall questionnaire [22]. The ICC coefficient was 0.932 (CI 0.919, 0.937, *p* < 0.001) indicating excellent reliability [22].

### Analysis

Data was extracted from the REDCap® database. Descriptive statistics on the characteristics of caregivers and care recipients were tabulated. Means with standard deviations, or medians with interquartile ranges were calculated for continuous variables, while frequencies and percentages were calculated for categorical variables. Association between Clinical Frailty Status and Caregiver Burden was analysed with Chi-Square Test. Post-hoc Bonferroni tests were performed to examine if the difference between groups was statistically significant for multiple comparisons, where necessary. Spearman’s rank correlation coefficient was used to determine the relationship of the number of chronic conditions that a care recipient has and ZBI. Multivariable analysis using logistic regression was used to investigate the factors affecting caregiver burden. Data was analysed using Statistical Package for Social sciences (SPSS) version 24 with the significance level set at *p* < 0.05 (two-tailed).

## Results

### Caregivers

A total of 205 eligible caregivers were approached, of whom 188 agreed to participate, giving a response rate of 91.7%. The characteristics of the caregivers are shown in Table 1. Their median age was 62.0 years (IQR 52.0–70.0). Almost two-thirds were female and 70.2% of them were married. 54.3% of them were working, 59.6% were caregivers to their parents and 18.1% of them had multimorbidity. The presence of an alternative caregiver was available to 61.2% of caregivers.

**Table 1:** Characteristics of the Caregivers

Characteristics of the caregivers	N (%)	Burden Perceived N(%)	***p*** value
**Age, yrs**			
*Median (IQR)*	62.0 (52.0–70.0)		0.382
**Gender**			
Male	66 (35.1)	48(72.7)	0.585
Female	122 (64.9)	87(71.1)	
**Ethnicity**			
Chinese	158 (84.0)	118(74.7)	0.117
Non-Chinese	30 (16.0)	16 (53.3)	
**Marital Status**			
Married	132 (70.2)	99(75.0)	0.667
Not Married	56 (29.8)	39(69.6)	
**Main work status, over the last 12 months**			
Working	102 (54.3)	74(72.5)	0.222
Not working	86 (45.7)	57(66.3)	
**Relationship with care recipient**			
Child	112 (59.6)	80(71.4)	
Spouse	50 (26.6)	35(70.0)	0.702
Others	26 (13.8)	8(30.8)	
**Number of chronic conditions**			
No chronic conditions	65 (34.6)	46(70.8)	
1 or 2 conditions	89 (47.3)	61(68.5)	0.305
3 or more conditions	34 (18.1)	29(85.3)	
**Presence of Alternative Caregivers**			
Yes	115 (61.2)	84(73.0)	0.637
No	73 (38.8)	51(69.8)	
**Duration of Caregiving, yrs**			
*Median (IQR)*	5.0 (3.0–10.0)		0.896
**Time Spent Caregiving per week, hrs**			
*Median (IQR)*	20.0 (12.0–30.0)		0.014
**Zarit Burden Index**			
*Median (IQR)*	15.0 (9.0–22.0)		
No burden perceived	53 (28.2)		
Burden perceived (ZBI 12 score ≥ 10)	135 (71.8)		

The caregivers interviewed were involved in care for a median of 5.0 years (IQR 3.0–10.0). and spent a median of 20.0 h (IQR 12.0–30.0) per week on caregiving work. They reported a median score of 15.0 (IQR 9.0–22.0) on the ZBI. A total of 71.8% of caregivers perceived burden based on the ZBI.

### Care recipients

The characteristics of the care recipients were described in Table 2. The 188 care recipients had a median age of 81.0 years (IQR 75.0–86.0). Dementia was present in 33.5% of them. The number of chronic conditions that each care recipient had was 4.0 (IQR 4.0–5.0).

**Table 2:** Characteristics of the Care Recipients

Characteristics of Care Recipient	N (%)	Burden Perceived N(%)	***p*** value
**Age Group**			
*Median (IQR)*	81.0 (75.0–86.0)		0.737
**Clinical Frailty Scale (CFS)**			
CFS 4 (Vulnerable)	22 (11.7)	14 (63.6)	
CFS 5 (Mildly Frail)	59 (31.3)	35 (59.3)	
CFS 6 (Moderately Frail)	49 (26.1)	40 (81.6)	0.03
CFS 7 (Severely Frail)	58 (30.9)	46 (79.3)	
**Presence of Dementia in Care Recipient**			
No	125 (66.5)	87(69.6)	0.794
Yes	63 (33.5)	48(76.2)	
**Number of chronic conditions**			
*Median (IQR)*	4.0 (4.0–5.0)		

The Chi-square test showed that there was a statistically significant difference between the different CFS groups for caregivers’ perceived burden (*p* = 0.03). This was before Bonferroni correction.

Table 3 shows that the proportion of caregiver burden perceived was statistically significant different between CFS 5 & CFS 6 and CFS 5 & CFS 7 using a *p* value of 0.05 as the cut-off. However, there were no statistically significant differences between the different CFS groups for the proportion of burden perceived by the caregivers after Bonferroni correction [23].

**Table 3:** Bonferroni Correction

Bonferroni Correction	***p*** value
CFS4 & CFS5	0.80
CFS4 & CFS6	0.13
CFS4 & CFS7	0.16
CFS5 & CFS6	0.02
CFS5 & CFS7	0.03
CFS6 & CFS7	0.81

Null hypothesis was rejected if *p* < (0.05/6 = 0.0083)

An analysis to investigate the correlation between the number of chronic conditions that a care recipient had and the Zarit Burden Index was performed. The Spearman’s rank correlation coefficient was 0.16 (*p* = 0.03).

Stratification analysis done showed that the time spent caregiving per week was significantly different among patients with different CFS scores (*p* = 0.04). There was no significant difference in the time spent caregiving per week between care recipients with dementia and without (*p* = 0.35).

Multivariable logistic regression analysis (Table 4) was used to analyse factors associated with caregivers’ perceived burden. A cut-off score of 10 or above on the ZBI indicated that the family caregiver perceived burden. After adjustment, the time spent caregiving per week was associated independently with primary family caregivers’ perception of burden (OR 1.04, 95%CI: 1.01,1.08, *P* = 0.01). We also found that non-Chinese primary family caregivers when compared to Chinese primary family caregivers had less perceived burden (OR 0.34, 95%CI: 0.13,0.93, *P* = 0.03).

**Table 4:** Multivariable Analysis

Variable (Caregiver)	Adjusted OR	95% C.I.		Sig.	Variable (Care Recipient)	Adjusted OR	95% C.I.		Sig.
		Lower	Upper				Lower	Upper	
**Age of the Caregiver**	0.98	0.94	1.03	0.37	**Age of care recipient**	0.97	0.92	1.03	0.37
**Gender**					**Clinical Frailty Scale (CFS) of care recipient**				
Male	1				CFS 4 (Vulnerable)	1			
Female	0.83	0.38	1.83	0.65	CFS 5 (Mildly Frail)	0.64	0.19	2.15	0.47
					CFS 6 (Moderately Frail)	2.13	0.52	8.74	0.29
**Ethnicity**					CFS 7 (Severely Frail)	1.43	0.36	5.63	0.61
Chinese	1								
Non-Chinese	0.34	0.13	0.93	***0.03***	**Presence of Dementia in Care Recipient**				
					No	1			
**Marital Status**					Yes	0.80	0.35	1.80	0.59
Married	1								
Not Married	0.41	0.16	1.04	0.06	**Number of chronic conditions in care recipient**	1.36	0.98	1.89	0.06
**Caregiver’s main work status, over the last 12 months**									
Working	1								
Not working	0.63	0.25	1.56	0.32					
**Relationship with care recipient**									
Child	1								
Spouse	0.36	0.09	1.49	0.16					
Others	1.57	0.47	5.24	0.46					
**Number of chronic conditions in caregiver**									
No chronic conditions	1								
1 or 2 conditions	1.02	0.40	2.60	0.98					
3 or more conditions	2.22	0.58	8.58	0.25					
**Presence of alternative caregivers**	1.46	0.57	3.72	0.43					
**Duration of caregiving, yrs**	0.97	0.90	1.04	0.34					
**Time spent caregiving per week, hrs**	1.04	1.01	1.08	***0.01***					

## Discussion

Our finding of 71.8% of family caregivers of frail older adults with multimorbidity experiencing caregiver burden was higher than similar studies conducted overseas where about 60% of informal caregivers perceived burden [13, 17]. The higher percentage of our local caregivers perceiving burden may be explained due to societal norms with emphasis on filial piety and family members being involved in caregiving duties [24]. In addition, urbanisation has produced increasingly smaller families, thus reducing the number of available family caregivers and limiting the extent to which the burden can be shared between family members [25].

In our study, bivariate analysis in Table 2 showed an association between the frailty status of care recipients and the burden perceived by their respective primary family caregivers (*P* = 0.03). Table 3 showed the Bonferroni correction that was performed to see which of the comparisons were statistically significantly different from each other after adjusting the *p* value to account for multiple comparisons. Interestingly, none of the comparisons was found to be statistically significant after the correction. The Bonferroni correction is commonly employed to reduce type I error (i.e., rejecting the null hypothesis when the null hypothesis is true) when multiple comparisons were conducted [26]. However, using the Bonferroni correction may be too conservative and resulted in type II error (i.e. accepting the null hypothesis when the null hypothesis is false). Furthermore, type I errors cannot be decreased (the aim of Bonferroni adjustments) without inflating type II errors (the probability of accepting the null hypothesis when the alternative is true) [27]. For this instance, we disregarded the use of the Bonferroni Correction and accepted the association between the frailty status of care recipients and the burden perceived by their primary caregivers as significant. However, when the different levels of frailty status were included in the multivariable logistic regression after adjusting for other factors, the frailty status of care recipient was not associated with caregiver burden anymore. Similarly, a study by Aggar et al. found that caregivers of care recipients deemed severely frail (Fried Frailty Status > 3) did not differ from caregivers of care recipients deemed mildly frail (Fried Frailty Status = 3) in terms of time demands and self-esteem [28, 29].

There was a very weak correlation between the number of chronic conditions care recipients had and caregivers’ perception of burden on bivariate analysis. The Spearman’s rank correlation coefficient was 0.16 (*P* = 0.03). When this variable was put into the multivariable logistic regression, the odds for the increase in the number of chronic conditions that care recipients had was positively associated with caregiver burden (OR 1.36, 95%CI: 0.98,1.89, *P* = 0.06) but this was not statistically significant. One possible explanation could be that a proportion of our study population had stable common chronic conditions, for example hypertension or hyperlipidaemia which did not contribute to caregiver burden significantly. A study in Egypt on 186 family caregivers of older adults also found no significant association between care recipients’ number of chronic diseases and caregivers’ burden [30].

Notably, 33.5% of care recipients in our study had dementia. Although it has been shown that care recipients with dementia increases caregiver burden [31, 32], our study showed otherwise. We did not find a significant association between the presence of dementia and caregivers’ perceived burden. This could be because care recipients who had dementia in our study were early or mild cases whereas those in the literature had significant numbers who exhibited behavioural and psychological symptoms of dementia.

One of the findings from our study was that more time spent caregiving per week was associated with caregivers’ perception of burden. This finding is similar to other studies. A cross-sectional study of 200 community residing patients in China, showed that longer hours of caregiving corresponded with an increase in caregiver burden experienced [33]. Additionally, other studies done in Turkey and the Netherlands reported that caregivers who invested more time in caregiving had increased worry and higher burden [34, 35].

The other finding in our study was that ethnicity of the caregiver was an independent factor that was associated with caregiver burden amongst primary family caregivers of frail older adults with multimorbidity. Specifically, in our study, Chinese caregivers had almost three times the odds of perceiving burden when compared to the non-Chinese caregivers. This is similar to a study of 385 caregivers of older people who attended a community clinic in Malaysia, which found ethnicity to be an independent factor that was associated with caregivers who were burdened [36]. In this study and another, also in Malaysia, a country with a multi-ethnic composition, Chinese caregivers were found to have a higher level of burden [36, 37]. A possible explanation for this finding could be because in the Chinese family, the obligation to care for a dependent elder is primarily influenced by cultural values of family responsibility [ 38] and filial obligation as one of the potential motivating factors in a caregiving relationship [39]. In Taiwan, which has a largely homogenous Chinese population, coupled with the Chinese tradition of filial piety, caregiver burden has become a pervasive problem in Taiwanese people, especially women, who are expected to assume the role of primary caregiver [40]. Likewise, in Singapore where our study was conducted, the Chinese primary family caregivers may be burdened by similar concepts and values of family responsibility and obligation.

Finally, studies on the impact of relationship to care recipient on caregiver burden found mixed results. While one study by Oldenkamp et al. did not find the type of care relationship to be significant [35], others found that children caregivers, particularly daughters and daughters-in-law had higher burden [13, 41, 42]. Our study did not find an association between relationship to care recipient and caregiver burden. Our study also did not find any association between gender of caregiver and caregiver burden, which is consistent with current literature [43].

### Strengths of the study

This is the first study conducted in Singapore looking at caregivers of frail older adults with multimorbidity. Reporting bias was minimised as this interviewer-administered study was carried out by a small team of three interviewers who have standardised the interview methods prior to the start of the research project.

### Limitations of the study

One of the limitations of our study is the use of convenience sampling. However, we minimised potential bias by inviting all eligible caregiver-care recipient dyads who attended the clinic during the recruitment period. There may also be non-response bias as the participants who consented to take part in the study may differ from those who do not. However, this is also perceived to be low as our response rate was high at 91.7%. Being a cross-sectional study, temporal associations between the independent and outcome variables cannot be made. Finally, there was limitation to the available characteristics of the caregivers and care recipients being studied.

## Conclusion

Our study showed that 71.8% of family caregivers of frail adults with multimorbidity experienced caregiver burden. After adjusting for other factors, being a Chinese primary family caregiver compared to non-Chinese ethnic groups as well as being a primary family caregiver who spent increased time caregiving per week were the two factors positively associated with family caregiver burden.

We suggest that further exploratory studies to find out the reasons for Chinese primary family caregivers being more burdened compared to the non-Chinese primary family caregivers as well as the specific factors related to increased time caregiving per week and primary family caregiver burden. This would help in the development of specific interventions with the goal of alleviating caregiving burden.

## Data Availability

All data generated or analysed during this study are included in this published article.
